# Hopeahainol C monohydrate

**DOI:** 10.1107/S1600536811017053

**Published:** 2011-05-14

**Authors:** Hoong-Kun Fun, Kanokorn Sudto, Hui-Ming Ge, Ren-Xiang Tan, Supa Hannongbua, Suchada Chantrapromma

**Affiliations:** a X-ray Crystallography Unit, School of Physics, Universiti Sains Malaysia, 11800 USM, Penang, Malaysia; b Department of Chemistry, Faculty of Science, Kasetsart University, Jatujak, Bangkok 10900, Thailand; c Institute of Functional Biomolecules, State Key Laboratory of Pharmaceutical Biotechnology, Nanjing University, Nanjing 210093, People’s Republic of China; d Crystal Materials Research Unit, Department of Chemistry, Faculty of Science, Prince of Songkla University, Hat-Yai, Songkhla 90112, Thailand

## Abstract

In the structure of the title compound, C_28_H_16_O_6_·H_2_O [systematic name 3,11-bis(4-hydroxyphenyl)-4,12-dioxapentacyclo[8.6.1.1^2,5^.0^13,17^.0^9,18^]octadeca-1(16),2,5(18),6,8,10,13(17),14-octaene-7,15-diol monohydrate], the hopeahainol C mol­ecule lies about an inversion center with the solvent water mol­ecule located on a crystallographic twofold axis. Hopeahainol C is an oligostillbenoid compound and was isolated from the bark of *Shorea roxburghii* G. Don. The five central fused rings are essentially planar with an r.m.s. deviation of 0.0173 (3) Å. The 4-hy­droxy­phenyl ring is twisted with respect to this plane, with the dihedral angle between the phenyl ring and the fused-ring system being 41.70 (10)°. The crystal features inter­molecular O—H⋯O hydrogen bonds. These inter­actions link the hopeahainol C mol­ecules into chains along the *b* axis. Water mol­ecules are located inter­stitially between the hopeahainol C mol­ecules linked by O(water)—H⋯O(hy­droxy) and O(hy­droxy)—H⋯O(water) hydrogen bonds. π–π inter­actions are also observed with centroid–centroid distances of 3.6056 (17) and 3.5622 (17) Å. Short O⋯O contacts [2.703 (2)–2.720 (3) Å] are also present in the crystal.

## Related literature

For bond-length data, see: Allen *et al.* (1987 ▶). For background to oligostillbenoids and their activities, see: Cai *et al.* (2003 ▶); Donnelly *et al.* (2004 ▶); Ge *et al.* (2009 ▶); Jang & Pezzuto (1999 ▶); Stivala *et al.* (2001 ▶). For details of Dipterocarpaceae plants, see: Gorham (1995 ▶); Hakim (2002 ▶); Sotheeswaran & Pasuphaty (1993 ▶); Symington (1974 ▶). For the stability of the temperature controller used in the data collection, see Cosier & Glazer, (1986 ▶). 
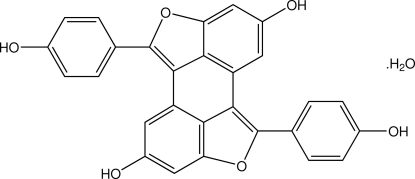


## Experimental

### 

#### Crystal data

C_28_H_16_O_6_·H_2_O
*M* *_r_* = 466.42
Monoclinic, 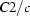

*a* = 21.225 (4) Å
*b* = 3.8500 (7) Å
*c* = 25.353 (5) Å
β = 108.933 (4)°
*V* = 1959.7 (6) Å^3^
*Z* = 4
Mo *K*α radiation
μ = 0.11 mm^−1^
*T* = 100 K
0.25 × 0.15 × 0.05 mm

#### Data collection

Bruker APEX DUO CCD area-detector diffractometer
Absorption correction: multi-scan (*SADABS*; Bruker, 2009 ▶) *T*_min_ = 0.972, *T*_max_ = 0.994
7974 measured reflections
2171 independent reflections
1463 reflections with *I* > 2σ(*I*)
*R*_int_ = 0.082

#### Refinement

*R*[*F* ^2^ > 2σ(*F* ^2^)] = 0.068
*wR*(*F* ^2^) = 0.161
*S* = 1.07
2171 reflections
163 parameters
H atoms treated by a mixture of independent and constrained refinement
Δρ_max_ = 0.27 e Å^−3^
Δρ_min_ = −0.35 e Å^−3^

### 

Data collection: *APEX2* (Bruker, 2009 ▶); cell refinement: *SAINT* (Bruker, 2009 ▶); data reduction: *SAINT*; program(s) used to solve structure: *SHELXTL* (Sheldrick, 2008 ▶); program(s) used to refine structure: *SHELXTL*; molecular graphics: *SHELXTL*; software used to prepare material for publication: *SHELXTL* and *PLATON* (Spek, 2009 ▶).

## Supplementary Material

Crystal structure: contains datablocks global, I. DOI: 10.1107/S1600536811017053/sj5138sup1.cif

Structure factors: contains datablocks I. DOI: 10.1107/S1600536811017053/sj5138Isup2.hkl

Supplementary material file. DOI: 10.1107/S1600536811017053/sj5138Isup3.cml

Additional supplementary materials:  crystallographic information; 3D view; checkCIF report

## Figures and Tables

**Table 1:** Hydrogen-bond geometry (Å, °)

*D*—H⋯*A*	*D*—H	H⋯*A*	*D*⋯*A*	*D*—H⋯*A*
O1*W*—H1*W*1⋯O2^i^	0.92 (3)	1.83 (3)	2.720 (2)	163 (3)
O3—H3*A*⋯O1*W*^ii^	0.82	1.89	2.703 (2)	169
O2—H2*A*⋯O3^iii^	0.82	2.00	2.716 (3)	145

Symmetry codes: (i) 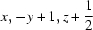; (ii) 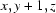; (iii) 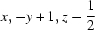.

**Fig. 1.**
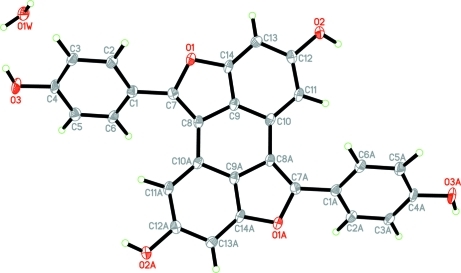
The molecular structure of the title compound, with 60% probability displacement ellipsoids and the atom-numbering scheme. Atoms with suffix A were generated by the symmetry code 1/2-x, 3/2-y, -z.

**Fig. 2.**
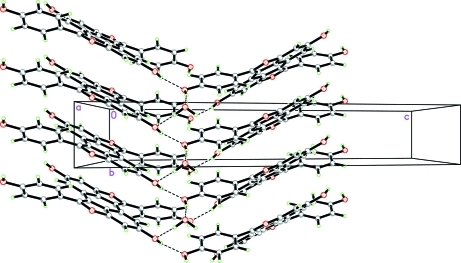
The crystal packing of the title compound viewed down the a axis, showing chains running along the b axis. Hydrogen bonds are shown as dashed lines.

## supplementary crystallographic information

### Comment

The genus *Shorea* is the largest genus of the family Dipterocarpaceae and is mostly distributed in Southeast Asia (Symington, 1974). The Dipterocarpaceous plant has already proved to be a rich source of oligostilbene compounds that are derived from stilbene and resveratrol (3,5,4'-trihydroxystilbene) (Gorham, 1995; Hakim, 2002; Sotheeswaran & Pasuphaty, 1993). It also has been known that resveratrol possesses various biological activities including antioxidant (Cai *et al*., 2003), anti-cancer, chemo-preventive (Jang & Pezzuto, 1999; Stivala *et al.*, 2001) and anti-inflammatory properties (Donnelly *et al.*, 2004). During the course of our research on searching for novel bioactive compounds from Thai dipeterocarpaceous plants, the title compound (I), known as hopeahainol C, was obtained from *Shorea roxburghii* G. Don. Hopeahainol C is an oligostilbenoid, which is a highly unsaturated resveratrol dimer, and it possesses potent antioxidant activity (Ge *et al.*, 2009). Herein we report its crystal structure.

The molecule of the title oligostilbenoid (I) (Fig. 1), C_28_H_16_O_6_.H_2_O, is a symmetrical dimer. Its asymmetric unit contains one half-molecule. The complete molecule of hopeahainol C is generated by a crystallographic center of symmetry 1/2-x, 3/2-y, -z whereas the other hydrogen atom of the water molecule is generated by a two-fold rotation axis -x, y, 1/2-z. The five central fused rings are essentially planar with the *r.m.s*. 0.0173 (3) Å for the eighteen non-hydrogen atoms. The 4-hydroxyphenyl ring is twisted which respect to the five central fused rings with the dihedral angle between the phenyl and the five central fused rings being 41.65 (10)°. The dihedral angle between the phenyl and the attached dihydrofuran (O1/C7–C9/C14) rings is 40.50 (15)°. The two hydroxy groups of the half molecule are co-planar with the attached benzene ring with the torsion angles O3–C4–C5–C6 = -178.1 (2)° and C10–C11–C12–O2 = -179.5 (2)°. The bond distances are of normal values (Allen *et al.*, 1987).

In the crystal packing (Fig. 2), the molecules of hopeahainol C are linked into chains along the *b* axis by O(hydroxy)—H···O(hydroxy) hydrogen bonds which form between the two hydroxy groups (Table 1). The water molecules are located in the interstitials of hopeahainol C molecules and are linked to the molecules of hopehainol C by two types of hydrogen bond *i.e*. O(water)—H···O(hydroxy) and O(hydroxy)—H···O(water) hydrogens bond (Table 1). The crystal is consolidated by these O—H···O hydrogen bonds. π–π interaction with Cg_1_···Cg_3_ distance = 3.6055 (17) Å (symmetry code: x, 1+y, z) and Cg_2_···Cg_3_ distance = 3.5622 (17) Å (symmetry code: 1/2-x, 5/2-y, -z) were observed where *Cg*1, *Cg*2 and *Cg*3 are the centroids of the O1/C7–C9/C14, C8–C10/C8A–C10A and C9–C14 rings, respectively. In addition O···O short contacts [2.703 (2)-2.720 (3) Å] are also presented in the crystal.

### Experimental

The dried powdered bark of *Shorea roxburghii* G. Don. (1 kg) which was collected during June-August 2010 from Sam Sung District, Khon Kaen province in the northeastern part of Thailand, was macerated in C_2_H_5_OH (2.5 L) for 7 days. The slurry was filtered and the ethanolic extract obtained was dried with a rotary evaporator under reduced pressure at 313 K. The dried extract (98 g) was ground, dissolved in CH_3_OH, mixed with silica gel (118 g), and then dried in hot air oven at 333 K for one day. The sample was separated by using a wet column chromatographic technique. The column was packed with 1 kg of silica gel (200-300 mesh) and was eluted with gradient mixtures of CHCl_3_ and CH_3_OH (100:0 to 0:100), to give 8 major fractions (A–H). Fraction G was further isolated using  sephadex column chromatography, eluted with 100% CH_3_OH. Only the blue methanolic extract could be selectively collected and was pre-concentrated and heated for 5-10 minutes at 313-333 K before being left for 2-3 days at 298 K to allow crystallization of hopeahainol C. Colorless needle-shaped single crystals of the hopeahainol C suitable for *X*-ray structure determination were obtained from CH_3_OH by slow evaporation at room temperature after a few days.

### Refinement

The water H atom was located in a difference map and refined isotropically. The remaining H atoms were placed in calculated positions with d(O—H) = 0.82 Å and d(C—H) = 0.93 Å for aromatic. The *U*_iso_ values were constrained to be 1.5*U*_eq_ of the carrier atom for hydroxy and 1.2*U*_eq_ for the remaining H atoms. The highest residual electron density peak is located at 0.66 Å from C9 and the deepest hole is located at 0.90 Å from C4.

### Crystal data

**Table:**

C_28_H_16_O_6_·H_2_O	*F*(000) = 968
*M**_r_* = 466.42	*D*_x_ = 1.581 Mg m^−^^3^
Monoclinic, *C*2/*c*	Mo *K*α radiation, λ = 0.71073 Å
Hall symbol: -C 2yc	Cell parameters from 2171 reflections
*a* = 21.225 (4) Å	θ = 2.0–24.8°
*b* = 3.8500 (7) Å	µ = 0.11 mm^−^^1^
*c* = 25.353 (5) Å	*T* = 100 K
β = 108.933 (4)°	Needle, colorless
*V* = 1959.7 (6) Å^3^	0.25 × 0.15 × 0.05 mm
*Z* = 4	

### Data collection

**Table:**

Bruker APEX DUO CCD area-detector diffractometer	2171 independent reflections
Radiation source: sealed tube	1463 reflections with *I* > 2σ(*I*)
graphite	*R*_int_ = 0.082
φ and ω scans	θ_max_ = 27.5°, θ_min_ = 2.0°
Absorption correction: multi-scan (*SADABS*; Bruker, 2009)	*h* = −27→27
*T*_min_ = 0.972, *T*_max_ = 0.994	*k* = −4→4
7974 measured reflections	*l* = −32→32

### Refinement

**Table:**

Refinement on *F*^2^	Primary atom site location: structure-invariant direct methods
Least-squares matrix: full	Secondary atom site location: difference Fourier map
*R*[*F*^2^ > 2σ(*F*^2^)] = 0.068	Hydrogen site location: inferred from neighbouring sites
*wR*(*F*^2^) = 0.161	H atoms treated by a mixture of independent and constrained refinement
*S* = 1.07	*w* = 1/[σ^2^(*F*_o_^2^) + (0.0792*P*)^2^ + 1.9771*P*] where *P* = (*F*_o_^2^ + 2*F*_c_^2^)/3
2171 reflections	(Δ/σ)_max_ = 0.001
163 parameters	Δρ_max_ = 0.27 e Å^−^^3^
0 restraints	Δρ_min_ = −0.35 e Å^−^^3^

### Special details

**Table:**

Experimental. The crystal was placed in the cold stream of an Oxford Cryosystems Cobra open-flow nitrogen cryostat (Cosier & Glazer, 1986) operating at 100.0 (1) K.
Geometry. All esds (except the esd in the dihedral angle between two l.s. planes) are estimated using the full covariance matrix. The cell esds are taken into account individually in the estimation of esds in distances, angles and torsion angles; correlations between esds in cell parameters are only used when they are defined by crystal symmetry. An approximate (isotropic) treatment of cell esds is used for estimating esds involving l.s. planes.
Refinement. Refinement of F^2^ against ALL reflections. The weighted R-factor wR and goodness of fit S are based on F^2^, conventional R-factors R are based on F, with F set to zero for negative F^2^. The threshold expression of F^2^ > 2sigma(F^2^) is used only for calculating R-factors(gt) etc. and is not relevant to the choice of reflections for refinement. R-factors based on F^2^ are statistically about twice as large as those based on F, and R- factors based on ALL data will be even larger.

### Fractional atomic coordinates and isotropic or equivalent isotropic displacement parameters (Å^2^)

**Table:**

	*x*	*y*	*z*	*U*_iso_*/*U*_eq_	
O1W	0.0000	0.5114 (9)	0.2500	0.0199 (7)	
H1W1	0.0200 (15)	0.649 (9)	0.2803 (13)	0.036 (10)*	
O1	0.10067 (8)	0.6569 (5)	0.01488 (7)	0.0151 (5)	
O2	0.08243 (8)	0.1345 (5)	−0.16160 (7)	0.0170 (5)	
H2A	0.1076	0.0687	−0.1782	0.026*	
O3	0.11424 (8)	1.1827 (5)	0.25471 (7)	0.0193 (5)	
H3A	0.0782	1.2799	0.2487	0.029*	
C1	0.14502 (11)	0.9053 (7)	0.10634 (10)	0.0136 (6)	
C2	0.08471 (11)	1.0602 (8)	0.10499 (10)	0.0156 (6)	
H2B	0.0521	1.1031	0.0709	0.019*	
C3	0.07334 (12)	1.1499 (7)	0.15414 (10)	0.0155 (6)	
H3B	0.0331	1.2504	0.1530	0.019*	
C4	0.12224 (12)	1.0890 (8)	0.20487 (10)	0.0156 (6)	
C5	0.18124 (12)	0.9313 (8)	0.20726 (10)	0.0165 (6)	
H5A	0.2133	0.8855	0.2415	0.020*	
C6	0.19264 (12)	0.8408 (8)	0.15796 (10)	0.0152 (6)	
H6A	0.2327	0.7357	0.1595	0.018*	
C7	0.15626 (11)	0.8033 (7)	0.05476 (10)	0.0136 (6)	
C8	0.20988 (11)	0.8046 (8)	0.03621 (10)	0.0130 (6)	
C9	0.18721 (11)	0.6449 (7)	−0.01786 (10)	0.0124 (6)	
C10	0.22099 (11)	0.5734 (7)	−0.05605 (10)	0.0119 (6)	
C11	0.18493 (12)	0.4004 (7)	−0.10467 (10)	0.0150 (6)	
H11A	0.2051	0.3459	−0.1312	0.018*	
C12	0.11835 (12)	0.3081 (7)	−0.11382 (10)	0.0137 (6)	
C13	0.08421 (11)	0.3852 (7)	−0.07681 (10)	0.0140 (6)	
H13A	0.0397	0.3262	−0.0838	0.017*	
C14	0.12110 (11)	0.5554 (7)	−0.02905 (10)	0.0129 (6)	

### Atomic displacement parameters (Å^2^)

**Table:**

	*U*^11^	*U*^22^	*U*^33^	*U*^12^	*U*^13^	*U*^23^
O1W	0.0212 (12)	0.026 (2)	0.0148 (13)	0.000	0.0088 (11)	0.000
O1	0.0162 (8)	0.0197 (12)	0.0120 (8)	−0.0010 (8)	0.0081 (7)	−0.0009 (9)
O2	0.0175 (8)	0.0233 (13)	0.0119 (8)	−0.0030 (8)	0.0070 (7)	−0.0049 (9)
O3	0.0252 (9)	0.0244 (13)	0.0129 (9)	0.0047 (9)	0.0128 (7)	0.0005 (9)
C1	0.0164 (11)	0.0117 (16)	0.0152 (12)	−0.0029 (11)	0.0086 (9)	−0.0007 (12)
C2	0.0161 (11)	0.0175 (17)	0.0154 (12)	−0.0018 (11)	0.0079 (9)	0.0004 (12)
C3	0.0158 (11)	0.0165 (17)	0.0180 (12)	−0.0008 (11)	0.0106 (9)	−0.0006 (13)
C4	0.0217 (12)	0.0167 (17)	0.0129 (12)	−0.0037 (11)	0.0117 (10)	−0.0019 (12)
C5	0.0189 (11)	0.0174 (17)	0.0139 (12)	−0.0006 (11)	0.0064 (9)	0.0015 (12)
C6	0.0165 (11)	0.0155 (16)	0.0171 (12)	0.0000 (11)	0.0102 (9)	0.0002 (12)
C7	0.0157 (11)	0.0130 (16)	0.0120 (11)	−0.0012 (11)	0.0044 (9)	−0.0005 (12)
C8	0.0170 (11)	0.0128 (16)	0.0109 (11)	0.0002 (11)	0.0069 (9)	0.0009 (12)
C9	0.0166 (11)	0.0099 (15)	0.0122 (11)	0.0013 (11)	0.0068 (9)	0.0019 (12)
C10	0.0155 (11)	0.0102 (16)	0.0122 (11)	0.0019 (10)	0.0073 (9)	0.0035 (12)
C11	0.0198 (11)	0.0156 (17)	0.0128 (12)	0.0003 (11)	0.0097 (9)	0.0008 (12)
C12	0.0202 (11)	0.0100 (16)	0.0116 (11)	−0.0006 (11)	0.0060 (9)	0.0013 (12)
C13	0.0140 (10)	0.0140 (17)	0.0152 (12)	−0.0010 (11)	0.0065 (9)	0.0015 (12)
C14	0.0178 (11)	0.0114 (16)	0.0130 (12)	0.0010 (11)	0.0099 (9)	0.0017 (12)

### Geometric parameters (Å, °)

**Table:**

O1W—H1W1	0.92 (3)	C5—C6	1.392 (3)
O1—C14	1.377 (3)	C5—H5A	0.9300
O1—C7	1.399 (3)	C6—H6A	0.9300
O2—C12	1.377 (3)	C7—C8	1.365 (3)
O2—H2A	0.8200	C8—C9	1.436 (3)
O3—C4	1.377 (3)	C8—C10^i^	1.465 (3)
O3—H3A	0.8200	C9—C14	1.382 (3)
C1—C6	1.392 (3)	C9—C10	1.406 (3)
C1—C2	1.403 (3)	C10—C11	1.392 (3)
C1—C7	1.457 (3)	C10—C8^i^	1.465 (3)
C2—C3	1.388 (3)	C11—C12	1.402 (3)
C2—H2B	0.9300	C11—H11A	0.9300
C3—C4	1.386 (4)	C12—C13	1.391 (3)
C3—H3B	0.9300	C13—C14	1.376 (4)
C4—C5	1.375 (3)	C13—H13A	0.9300
			
C14—O1—C7	106.61 (17)	O1—C7—C1	114.32 (19)
C12—O2—H2A	109.5	C7—C8—C9	105.6 (2)
C4—O3—H3A	109.5	C7—C8—C10^i^	137.4 (2)
C6—C1—C2	118.5 (2)	C9—C8—C10^i^	116.94 (19)
C6—C1—C7	121.0 (2)	C14—C9—C10	121.3 (2)
C2—C1—C7	120.4 (2)	C14—C9—C8	107.8 (2)
C3—C2—C1	120.5 (2)	C10—C9—C8	130.8 (2)
C3—C2—H2B	119.8	C11—C10—C9	116.5 (2)
C1—C2—H2B	119.8	C11—C10—C8^i^	131.2 (2)
C4—C3—C2	119.7 (2)	C9—C10—C8^i^	112.2 (2)
C4—C3—H3B	120.2	C10—C11—C12	120.2 (2)
C2—C3—H3B	120.2	C10—C11—H11A	119.9
C5—C4—O3	117.2 (2)	C12—C11—H11A	119.9
C5—C4—C3	120.8 (2)	O2—C12—C13	115.8 (2)
O3—C4—C3	122.0 (2)	O2—C12—C11	120.6 (2)
C4—C5—C6	119.5 (2)	C13—C12—C11	123.5 (2)
C4—C5—H5A	120.3	C14—C13—C12	115.0 (2)
C6—C5—H5A	120.3	C14—C13—H13A	122.5
C1—C6—C5	121.0 (2)	C12—C13—H13A	122.5
C1—C6—H6A	119.5	C13—C14—O1	127.5 (2)
C5—C6—H6A	119.5	C13—C14—C9	123.3 (2)
C8—C7—O1	110.7 (2)	O1—C14—C9	109.2 (2)
C8—C7—C1	134.9 (2)		
			
C6—C1—C2—C3	0.7 (4)	C10^i^—C8—C9—C14	−178.9 (2)
C7—C1—C2—C3	178.5 (3)	C7—C8—C9—C10	−179.2 (3)
C1—C2—C3—C4	0.7 (4)	C10^i^—C8—C9—C10	2.0 (5)
C2—C3—C4—C5	−1.9 (4)	C14—C9—C10—C11	−1.7 (4)
C2—C3—C4—O3	178.0 (3)	C8—C9—C10—C11	177.4 (3)
O3—C4—C5—C6	−178.1 (2)	C14—C9—C10—C8^i^	179.0 (2)
C3—C4—C5—C6	1.8 (4)	C8—C9—C10—C8^i^	−1.9 (5)
C2—C1—C6—C5	−0.8 (4)	C9—C10—C11—C12	0.4 (4)
C7—C1—C6—C5	−178.5 (3)	C8^i^—C10—C11—C12	179.5 (3)
C4—C5—C6—C1	−0.5 (4)	C10—C11—C12—O2	−179.5 (2)
C14—O1—C7—C8	1.7 (3)	C10—C11—C12—C13	1.2 (4)
C14—O1—C7—C1	−176.0 (2)	O2—C12—C13—C14	179.3 (2)
C6—C1—C7—C8	−39.1 (5)	C11—C12—C13—C14	−1.3 (4)
C2—C1—C7—C8	143.2 (3)	C12—C13—C14—O1	−178.7 (3)
C6—C1—C7—O1	138.0 (3)	C12—C13—C14—C9	−0.1 (4)
C2—C1—C7—O1	−39.8 (4)	C7—O1—C14—C13	177.0 (3)
O1—C7—C8—C9	−1.0 (3)	C7—O1—C14—C9	−1.7 (3)
C1—C7—C8—C9	176.1 (3)	C10—C9—C14—C13	1.6 (4)
O1—C7—C8—C10^i^	177.4 (3)	C8—C9—C14—C13	−177.7 (3)
C1—C7—C8—C10^i^	−5.5 (6)	C10—C9—C14—O1	−179.6 (2)
C7—C8—C9—C14	−0.1 (3)	C8—C9—C14—O1	1.1 (3)

Symmetry codes: (i) −*x*+1/2, −*y*+3/2, −*z*.

### Hydrogen-bond geometry (Å, °)

**Table:**

*D*—H···*A*	*D*—H	H···*A*	*D*···*A*	*D*—H···*A*
O1W—H1W1···O2^ii^	0.92 (3)	1.83 (3)	2.720 (2)	163 (3)
O3—H3A···O1W^iii^	0.82	1.89	2.703 (2)	169
O2—H2A···O3^iv^	0.82	2.00	2.716 (3)	145

Symmetry codes: (ii) *x*, −*y*+1, *z*+1/2; (iii) *x*, *y*+1, *z*; (iv) *x*, −*y*+1, *z*−1/2.
